# CPT1A through succinylation regulates the CTCF/LINC01503‒91aa feedback loop to promote gemcitabine resistance in pancreatic ductal adenocarcinoma

**DOI:** 10.1002/ctm2.70776

**Published:** 2026-09-01

**Authors:** De‐Liang Fang, Si‐Yuan Lu, Zhi‐De Liu, Qiong‐Cong Xu, Yang‐Yinhui Yu, Ying‐Qin Zhu, Ming‐Jian Ma, Jing‐Yuan Ye, Xiao‐Yu Yin

**Affiliations:** ^1^ Department of Pancreato‐Biliary Surgery The First Affiliated Hospital of Sun Yat‐Sen University Guangzhou China

**Keywords:** chemoresistance, CPT1A, CTCF, fatty acid oxidation, LINC01503, succinylation

## Abstract

**Background:**

Pancreatic ductal adenocarcinoma (PDAC) is one of the most malignant solid tumors, characterized by strong invasiveness and poor clinical prognosis. Recent studies have revealed that long non‐coding RNAs (lncRNAs) can serve as translational templates to produce functional microproteins, which further participate in the regulation of cellular metabolism.

**Objective:**

To investigate the function and detailed mechanism of the novel protein 1503–91aa encoded by LINC01503 in gemcitabine‐resistant PDAC, and explore the possibility of clinical translation.

**Methods:**

The differentially expressed lncRNAs in gemcitabine‐resistant PDAC cells with coding potential were screened by whole transcriptome sequencing and coding prediction from database. A series of cellular, molecular and in vivo assays were performed to characterize the novel microprotein 1503–91aa, its upstream transcriptional regulation, and its role in mediating PDAC gemcitabine resistance. A series of molecular assays clarified the interaction between 1503–91aa and carnitine palmitoyl transferase 1A (CPT1A). Finally, the clinical translational potential of the CPT1A inhibitor etomoxir in combination with gemcitabine was validated using our center's gemcitabine‐resistant patient‐derived xenograft (PDX) model.

**Results:**

Here, we revealed that LINC01503 had coding potential in drug‐resistant PDAC and encoded a novel 91–amino acid protein, which designated as 1503–91aa. Simultaneously, LINC01503's transcriptional regulation is mediated by CTCF and then 1503–91aa expression levels were significantly upregulated in PDAC. Functionally, 1503–91aa, instead of LINC01503 confers gemcitabine resistance in vitro and in vivo. Mechanistically, 1503–91aa targeted the CPT1A, and up‐regulated CPT1A activity through antagonizing the association of malonyl‐CoA (MCoA), the best known metabolic intermediate inhibiting CPT1A, to promote fatty acid oxidation (FAO) and thereby facilitate gemcitabine resistance. We discovered that CPT1A functions as a succinyltransferase to regulate the succinylation level of CTCF, thereby controlling its stability. This establishes a positive feedback loop that continuously sustains 1503–91aa protein levels to regulate CPT1A activity. Consequently, this induces persistent FAO in PDAC cells, ensuring a continuous energy source that facilitates gemcitabine resistance in pancreatic cancer. Notably, the combined application of the etomoxir and gemcitabine exerts a synergistic effect on PDAC.

**Conclusions:**

These findings provide novel insights into the molecular mechanisms underlying gemcitabine resistance in PDAC and highlight CTCF/LINC01503–91aa/CPT1A feedback loop as a potential prognostic biomarkers and therapeutic targets.

**Key points:**

- We identified that LINC01503 is capable of encoding the protein product 1503–91aa.
- 1503–91aa competed with MCoA to unleash CPT1A activity for fatty acid oxidation and thereby mediating gemcitabine resistance in PDAC.
- Etomoxir and gemcitabine have synergistic effects in gemcitabine efficiency.

## Graphical abstract

LINC01503 encoded a 91‐amino acid protein named 1503‒91aa, which enhanced carnitine palmitoyl transferase 1A (CPT1A) activity and regulated fatty acid oxidation (FAO) by competitively binding to malonyl‐CoA. Meanwhile, we found a positive feedback loop whereby CPT1A acts as a succinyltransferase to stabilise CTCF (a transcription factor driving LINC01503 expression), and then maintain 1503‒91aa levels, which thereby sustaining persistent FAO and driving gemcitabine resistance in PDAC.

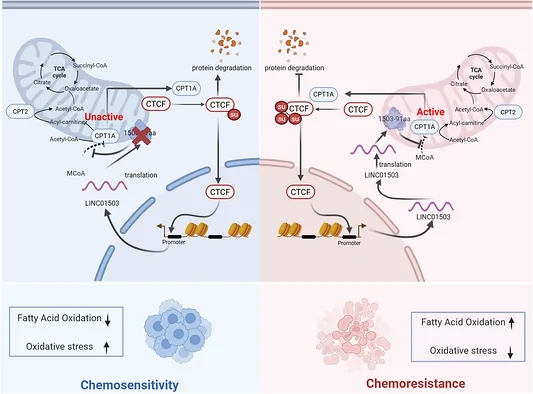

## 1 INTRODUCTION

Pancreatic ductal adenocarcinoma (PDAC) is one of the most malignant digestive cancers with rapid progression, dismal prognosis and overall 5‐year survival rate of less than 10%.^1^ Surgical radical resection is the only curable option and potential cure for PDAC. Regrettably, approximately 80% of patients with PDAC are no longer eligible for surgical intervention at the time of diagnosis with advanced disease.^2^ Gemcitabine (GEM)‐based combination chemotherapy strategies remain the primary therapeutic avenue for PDAC.^3^ And its primary mechanism of action is that GEM derivatives interfere with cellular DNA synthesis and block the cell cycle.^4^ However, owing to its inherent intratumoural heterogeneity and highly desmoplastic tumour microenvironment, the overall response rate of PDAC patients to GEM remains less than 20%.^5^, ^6^ Resistance of PDAC to chemotherapy is one of the most significant barriers that undermines therapeutic efficacy and leads to treatment failure. Therefore, it is of great importance to explore novel potential targets and therapeutic strategies for improving the sensitivity of PDAC to GEM.

Extensive reprogramming of metabolism in PDAC, as is the case for all cancers, supports tumour progression and therapeutic resistance.^7^, ^8^ The dysregulation of metabolism in cancer cells is a hallmark of malignant transformation.^9^ At a systemic level, the excessive energy hyperactivity of pancreatic cancer cells causes significant wasting of body composition and leads to cachexia.^10^ At the cellular level, this metabolic dysregulation is not limited to the classic Warburg effect; lipid metabolism is also profoundly upregulated.^11^ Fatty acids are fundamental cellular components and energy sources. Fatty acid oxidation (FAO) is a multistep process that breaks down long‐chain fatty acids to acetyl‐CoA, which is fully oxidised through the Krebs cycle and the electron transport chain to produce ATP.^12^ FAO replenishes the tricarboxylic acid (TCA) cycle and promotes NADPH and ATP production to overcome stress in cancer cells.^13^ Consequently, the capacity of pancreatic cancer cells to finely orchestrate this central FAO pathway is a likely key determinant in the development of resistance to GEM. Carnitine palmitoyl transferase 1A (CPT1A) is a key molecule located on the outer mitochondrial membrane that regulates the oxidative degradation of long‐chain fatty acids in the mitochondria for oxidative degradation.^14^ Recent studies have revealed that CPT1A has lysine succinyltransferase (LSTase) activity and promotes succinylation in cells.^15^, ^16^ Succinylation is the process of covalent binding of succinyl groups to the lysine residues of substrate proteins to affect their protein function.^17^ Succinylation can change the spatial structure and charged properties of substrate proteins and thus regulates various signaling pathways, including the TCA cycle, electron transport chain, glycolysis, FAO and urea cycle.^18^, ^19^

Recently, long non‐coding RNAs (lncRNAs) have been shown to participate in numerous physiological and pathological processes.^20^ Notably, recent progress in translatomics supports that lncRNAs exert crucial roles in the multiple biological processes mediated by their encoded products, which enriching the functional spectrum of lncRNAs.^21^, ^22^, ^23^ Mounting evidence has validated that dysregulated lncRNAs participate in tumour progression and metabolic/chemoresistance reprogramming across various malignancies. They can influence the expression of immune checkpoint molecules, thereby shape the tumour microenvironment and contribute to the immune evasion, and induce chemoresistance.^24^ Dysregulated transcription of lncRNAs mainly relies on key transcription factors binding their promoters to initiate transcription, with E2F1 exerting a central functional role here.^25^

To date, several lncRNAs have been identified that play important roles by encoding functional peptides in various malignancies, including PDAC, hepatocellular carcinoma, gastric cancer and glioblastoma.^26^, ^27^, ^28^, ^29^, ^30^ However, many lncRNAs with coding potential remain unidentified. In the present study, we investigated the unknown molecules and mechanisms of action in PDAC.

Here, we found that LINC01503 is capable of encoding the protein product 1503–91aa. The upregulation of 1503–91aa expression in GEM‐resistant pancreatic cancer is attributed to the transcriptional regulation of LINC01503 by CTCF. 1503–91aa, but not LINC01503, plays a critical role in the therapeutic effect of GEM on PDAC. Mechanistically, 1503–91aa played a critical role in FAO control by targeting CPT1A at Arg243. Furthermore, we found that CPT1A modulates CTCF transcriptional activity through succinylation. This establishes a positive feedback loop that consequently leads to a persistent increase in 1503–91aa protein levels. Our study also unveiled the synergistic effect of the CPT1A inhibitor etomoxir, disrupts this positive feedback loop, when combined with GEM in the treatment of PDAC. Our findings highlight the therapeutic potential of the positive feedback loop mediated by the lncRNA‐derived protein 1503–91aa as a target for GEM‐resistant PDAC.

## 2 MATERIALS AND METHODS

### 2.1 Patients’ specimens

All human samples were obtained from the First Affiliated Hospital of Sun Yat‐Sen University (FAHSYSU) in China from January 2016 to December 2020. The inclusion criteria included that the pathological diagnosis was PDAC; GEM was involved in the chemotherapy regimen; pathological tissue could be obtained; and there was no history of radiotherapy. A total of 45 paraffin‐embedded tissues from PDAC patients were collected before receiving GEM‐based chemotherapy. The inclusion criteria included: a pathological diagnosis of PDAC, no prior radiotherapy and GEM‐based chemotherapy. According to the Response Evaluation Criteria in Solid Tumours (RECIST1.1), 13 of the 45 patients were evaluated as GEM‐resistant (progressive disease), and three were evaluated as GEM‐sensitive (partial response). The human materials were obtained with the consent of patients and approved by the Medical Ethics Committee (IIT‐2021‐719).

### 2.2 Cell culture and transfection

The HEK‐293T (RRID: CVCL_0063), BxPC‐3 (RRID: CVCL_0186) and CFPAC‐1 (RRID: CVCL_1119) cell lines were purchased from the Cell Bank of the Chinese Academy of Science. Additionally, we performed short tandem repeat profiling to authenticate the cell lines. The results confirmed that the cell lines are correctly identified and free from contamination. HEK‐293T cells were cultured in dulbecco's modified eagle medium (DMEM, Gibco). BxPC‐3 and CFPAC‐1 cells were cultured in RPMI 1640 medium (Gibco). All media were supplemented with 10% foetal bovine serum (Gibco) in a humidified atmosphere of 5% CO_2_ at 37°C. No mycoplasma contamination was detected in all cells.

Wild‐type (WT) or mutant LINC01503 cDNAs with flanking sequences and ORFs were cloned into the pCDH‐CMV‐MCS‐EF1 vector by IGE Biotechnology. Coding sequences of 1503–91aa‐3×FLAG as well as full‐length and truncations of CPT1A were also inserted into the pCDH‐CMV‐MCS‐EF1 vector. Small guide RNA (sgRNA) and short hairpin RNA (shRNA) of LINC01503 were also purchased from IGE Biotechnology. A total of 3 × 10^5^ cells per well were seeded at in six‐well plates, and 3 µg of vector was transfected with 3 µL of DNA transfection reagent (Neofect). For stable transfection, cells were infected with the packaged lentivirus and selected with 2 µg/mL puromycin for 3 days. Surviving cells were then used to confirm efficiency.

### 2.3 Establishment of GEM‐resistant cell lines

WT PDAC cells, namely, BxPC‐3 and CFPAC‐1, were inoculated in the culture flask with a low initial concentration (100 nM) for 24 h. After drug treatment, the cells were cultured in drug‐free medium until their morphology and proliferation rate returned to normal. When returning to normal morphology and proliferation rate, the cells were treated with this concentration for 24 h again. This treatment process was repeated six times over a 6‐month period, with the GEM concentration incrementally increased to 1000 nM.

### 2.4 Animal experiments

For the tumourigenesis models, a total of 5 × 10^6^ BxPC‐3(gemcitabine‐resistant, GR) and CFPAC‐1(GR) cells were subcutaneously injected into the right armpits of 4‐week‐old male BALB/c nude mice. Following tumour formation, the mice were randomly divided into four groups and treatment commenced. GEM was administered intraperitoneally at a dose of 25 mg/kg body weight every 3 days for 24 consecutive days. After 4 weeks, the mice were sacrificed and tumours were isolated for volume calculations and weight measurements.

To create patient‐derived xenograft (PDX) models, surgical specimens from PDAC patients were cut into 3 mm^3^ pieces and engrafted into 5‐week‐old B‐NDG mice (Biocytogen), designated as generation 1 (P1). After several months of growth, PDX models of passages 2 (P2) and 3 (P3) were obtained. Small pieces of fresh P3 PDX tumour tissue were minced and transplanted subcutaneously into 4‐week‐old female BALB/c nude mice. Mice were randomly assigned to four treatment groups: phosphate buffered saline (PBS), etomoxir (40 mg/kg, intraperitoneally, once a week) and GEM (25 mg/kg, intraperitoneally, twice a week). At the end of the experiment, mice were sacrificed by asphyxiation with CO_2_, and the excised tumours were measured and fixed in 4% paraformaldehyde for further studies. The Institutional Review Board of the First Affiliated Hospital of Sun Yat‐Sen University approved all procedures (2024001324).

### 2.5 Reverse transcription and RT‐qPCR assay

Total RNA from tissues was extracted by RNAiso Plus (TaKaRa). Total RNA from cells was extracted by an RNA‐Quick Purification Kit (ESscience). RNA was reverse‐transcribed using an Evo M‐MLV RT Kit (Accurate Biology). qRT‐PCR assays were performed using 2×SYBR Green Pro Taq HS Premix II (Accurate Biology) according to the manufacturers’ protocols. All of the cDNA was amplified in a total of 40 cycles.

### 2.6 Immunoprecipitation

Cells were lysed with lysis buffer for Western blotting and immunoprecipitation (IP) (Beyotime) with protease and phosphatase inhibitors (Selleck). The supernatant was collected and incubated with primary antibodies at 4°C overnight. The lysates were then incubated with 30 µL of Protein A/G Magnetic Beads (Thermo Scientific) for 2 h at room temperature. The collected agarose‐protein was washed six times and then analysed in subsequent experiments.

### 2.7 Mass spectrometry

The total protein of the IP or protein separated by sodium dodecyl sulfate‑polyacrylamide gel electrophoresis (SDS‐PAGE) was used to perform MS analyses with a QExactive mass spectrometer (Thermo Scientific). The fragment spectra were analysed using the National Center for Biotechnology Information non‐redundant protein database with Mascot (Matrix Science). All MS analyses were conducted by Fitgene Biotech Co.

### 2.8 Western blot

Total proteins were extracted using cell lysis buffer for Western blotting and IP (Beyotime) with protease and phosphatase inhibitors (Selleck). Equal amounts of total proteins (30 µg) were separated by SDS‐PAGE and transferred onto polyvinylidene fluoride membranes (Merck Millipore). The membranes were incubated with the primary antibodies listed in Table S1 at 4°C overnight, followed by incubation with secondary antibodies (1:10 000; CST) at room temperature for 1 h. Signals were detected with Chemiluminescent HRP Substrate (Merck Millipore).

### 2.9 Immunohistochemistry

The immunostained sections were evaluated and scored independently by two pathologists. Paraffin sections were deparaffined, rehydrated, subjected to antigen retrieval and blocked for endogenous peroxidase activities. Sections were then incubated at 4°C overnight with the indicated antibodies (Table S1). The sections were then incubated with a horseradish peroxidase‐conjugated secondary antibody and DAB followed by counterstaining with haematoxylin. The anti‐Ki67 antibody was used at a dilution ratio of 1:5000. TUNEL staining was performed using the Fluorescein (FITC) Tunel Cell Apoptosis Detection Kit (Cat #G1501; Servicebio) following the manufacturer's protocol and optimisation procedures.

### 2.10 Immunofluorescence

PDAC cells (1 × 10^4^ cells) were fixed with 4% paraformaldehyde (Beyotime), permeabilised with .1% Triton X‐100 for 15 min, and incubated with primary antibodies at 4°C overnight (Table S1). After incubation with the secondary antibody and counterstaining with DAPI, the stained cells were observed with a confocal laser scanning microscope (Zeiss) and processed using ZEN imaging software.

### 2.11 Cell‐counting kit‐8 assay

Cell proliferation was evaluated using Cell‐Counting Kit‐8 (CCK‐8 Kit, Dojindo, CK04). Cells were seeded into 96‐well plates at the density of 2 × 10^4^ cells/well. At the indicated time points, 10 µL CCK‐8 was added into the medium of the cells, and optical density (OD) was measured spectrophotometer at a wavelength of 450 nm (Epoch, BioTek).

### 2.12 Colony formation

A total of 1.5 × 10^3^ cells per well were seeded into six‐well plates. After a week of growth, cells were fixed with 4% paraformaldehyde for 30 min and stained with .1% crystal violet (Beyotime) for another 30 min.

### 2.13 5‐Ethynyl‐2'‐deoxyuridine (EDU) assay

A total of 5 × 10^3^ cells were seeded in 96‐well plates and incubated with 50 µM EdU solution (C0075S, Beyotime). After 2 h of incubation, cells were fixed, washed and stained with fluorescent dye Apollo 567 and Hoechst 33342. The stained cells were observed with a confocal laser scanning microscope (Zeiss) and processed using ZEN imaging software.

### 2.14 Apoptosis assay

PDAC cells with different treatments were seeded into six‐well plate and cultured for 48 h. Then, the cells were collected, washed and stained by Annexin V‐APC/7‐AAD cell apoptosis assay kit (70‐AP105‐100, MultiSciences) according to the manufacturer's instruction. After staining, the apoptosis rate was measured by flow cytometer (Beckman Coulter).

### 2.15 BODIPY staining

The cells were cultured in a six‐well plate until they reached 60%–70% confluency. Then, the cells were treated as experiment requirements. The BODIPY dye was diluted in completely culture medium at 1:1000 concentration and incubated with cells for 30 min. The cells were then collected and centrifuged at 2000 rpm and washed twice with PBS. BODIPY staining was observed using was measured by flow cytometer (Beckman Coulter).

### 2.16 MitoSOX detection

PDAC cells seeded in plates were incubated with 10 µM MitoSOX Red (ThermoFisher) at 37°C for 20 min, washed 3× with serum‐free medium, and immediately analysed by flow cytometry.

### 2.17 Rate of oxygen consumption

Oxygen consumption rate (OCR) was measured with a Seahorse Bioscience XF‐24 extracellular analyser (Agilent) according to the protocol of the kit. Briefly, 50 000‒100 000 cells were seeded on Seahorse XF24 polystyrene culture plates in XF base medium containing 2 mM sodium pyruvate (P5280, Sigma‒Aldrich) and 4 mM glutamine (25030‐081, Gibco). OCR was measured by adding inhibitors at the indicated times with the follow concentrations: oligomycin (20 min, 1.0 mM), trifluoromethoxy carbonylcyanide phenylhydrazone (FCCP, 45 min, .5 mM) and Antimycin A/Rotenone (70 min, .5 mM). Three readings were taken after each injection, and the results were presented with pmol/min/well for OCR versus time (*x* axis).

### 2.18 Mitochondrial membrane potential measurement

Mitochondrial membrane potential (MMP) assay kit with 5,5′,6,6′‐tetrachloro‐1,1′,3,3′‐tetraethyl‐imidacarbocyanine iodide (JC‐1, C2005, Beyotime) was used to monitor and quantify MMP. PDAC cells were stained with 100 nM JC‐1 for 30 min at cell incubator, then images were taken by a fluorescence confocal microscope. The normalised intensity of the fluorescence of images was quantified by ImageJ software.

### 2.19 ATP measurement

PDAC cells were used for assay of cellular ATP, using an enhanced ATP detection kit (S0027, Beyotime) according to the instruction of the manufacturer. Chemiluminescence was read in Synergy2. A standard curve was used to calculate the ATP concentration in each sample, and the protein concentration was used to calibrate the ATP level.

### 2.20 Triglyceride assay

Triglyceride levels were measured by using Triglyceride Assay Kit (E1013‐105, Applygen) according to the instruction of manufacturer and normalised by protein concentration.

### 2.21 Statistical analysis

All statistical analyses were performed by GraphPad Prism version 8.0. Two‐tailed Student's *t*‐test and Wilcoxon signed‐rank test were used to compare two groups and one‐way analysis of variance (ANOVA) were used to compare multiple comparison correction. Kaplan‒Meier analysis was performed to calculate overall survival (OS). All in vitro cellular assays were repeated with at least three independent biological replicates; in vivo animal experiments were conducted using the indicated number of mice per group. The results are presented as the mean ± SD for parametric tests and as median ± interquartile range for non‐parametric tests. *p*‐Values < .05 were considered to indicate statistical significance.

## 3 RESULTS

### 3.1 Identification of functional peptides encoded by LINC01503 in GEM‐resistant PDAC

**FIGURE 1:**
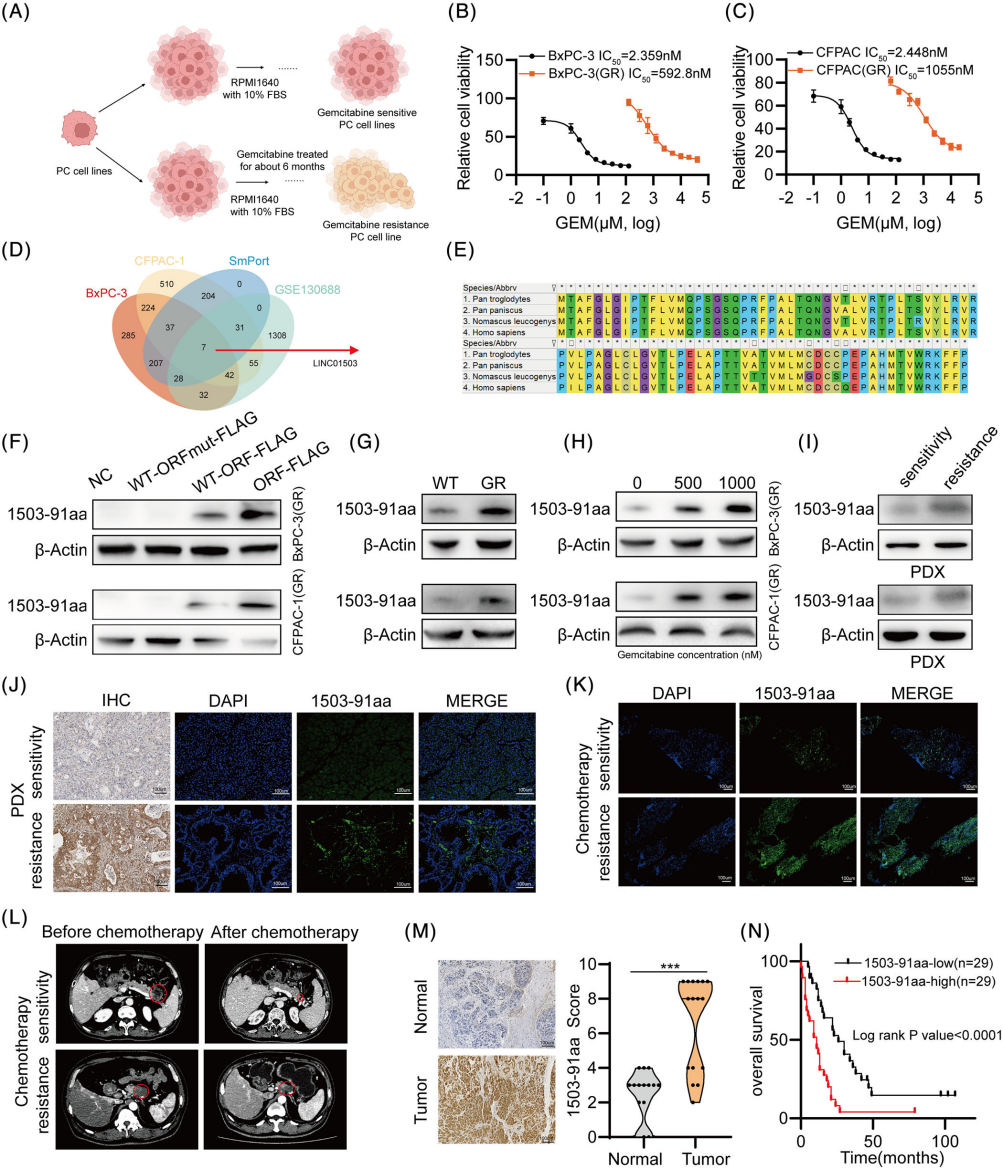
Identification and characterisation of 1503–91aa, a mitochondria‐localised protein encoded by LINC01503 that is upregulated in gemcitabine (GEM)‐resistant pancreatic cancer. (A) Schematic diagram illustrating the construction of GEM‐resistant pancreatic cancer cells. (B and C) Half‐maximal inhibitory concentration (IC_50_) values of GEM in BxPC‐3, BxPC‐3(gemcitabine‐resistant, GR), CFPAC‐1 and CFPAC‐1(GR) cells. (D) Strategy for identifying LINC01503 from GEM‐resistant long non‐coding RNA (lncRNA) genes that are associated with coding potential in pancreatic cancer. (E) The amino acid sequences of 1503–91aa among different species. (F) 1503–91aa protein expression transfected with wild‐type (WT) with ATG mutation, WT and 1503–91aa (ORF). (G) Determine the expression levels of 1503–91aa protein in GEM‐resistant cell lines. (H) Analysis of 1503–91aa protein levels in GEM‐treated BxPC‐3(GR) and CFPAC‐1(GR) cells at the indicated drug concentrations. (I) Detected the protein expression of 1503–91aa in GEM‐sensitive and GEM‐resistant. (J) Immunohistochemistry (IHC) and fluorescent assay (immunofluorescence, IF) as used to detect the expression of 1503–91aa protein. (K and L) Images of IF staining for 1503–91aa in GEM‐resistant and GEM‐sensitive tissues. Representative CT images illustrating GEM‐sensitive and GEM‐resistant pancreatic ductal adenocarcinoma (PDAC) tumours before and after GEM‐based chemotherapy. (M) Elevated 1503–91aa expression in PDAC tumour tissues compared to adjacent normal pancreatic tissues. (N) Negative correlation between 1503–91aa expression and the prognosis of PDAC patients. The data are shown as mean ± SD. ^*^ *p* < .05; ^**^ *p* < .01; ^***^ *p* < .001.

To investigate the mechanisms underlying GEM resistance in PDAC, we established GEM‐resistant PDAC cell lines by subjecting BxPC‐3/CFPAC‐1 cells to increasing doses of GEM (Figure 1A). After 6 months of GEM treatment, the half maximal inhibitory concentration (IC_50_) of GEM in BxPC‐3(GR) and CFPAC‐1(GR) cells was significantly higher than that in WT cells. Specifically, the IC_50_ values were 592.8 nm for BxPC‐3(GR) cells and 1055 nm for CFPAC‐1(GR) cells, compared with 2.359 and 2.448 nm for WT cells, respectively (Figure 1B,C). RNA sequencing was performed on both GEM‐resistant cell lines (BxPC‐3[GR] and CFPAC‐1[GR]) and their corresponding (WT) cells, this helped to identify differentially expressed lncRNAs. By combining the SmProt and GSE130688 PDAC databases for bioinformatics analysis, we obtained seven candidate genes (Figure 1D). Subsequently, we assessed the coding potential of lncRNAs and found that LINC01503 had the highest coding probability. Furthermore, as predicted by the ORF finder databases, LINC01503 begins translation at the start codon ATG and terminates at the stop codon TAA after a translation cycle. It might contain an ORF of 276 nt, which encodes a 91‐amino‐acid (91aa) protein; the sequence was also identified in the SmPort database (Figure S1A). Thus, we named the predicted protein 1503–91aa. Moreover, alignment analysis demonstrated that 1503–91aa is evolutionarily conserved among primates (Figure 1E). Subsequently, a 14‐amino‐acid (14‐aa) peptide within the specific sequence of the antigenic epitope was selected to generate a rabbit‐derived polyclonal antibody (Figure S1B). IP was performed to enrich endogenous 1503–91aa in BxPC‐3 cells, and the sequence of 1503–91aa was detected using mass spectrometry (MS) to further prove the existence of 1503–91aa in PDAC (Figure S1C). To further verify the presence of 1503–91aa in PDAC, we constructed FLGA‐tagged plasmids, which contained ORFs in LINC01503(WT) and 1503–91aa (ORF), and then we inserted an A base into the WT vector of LINC01503 to construct a code‐shifting mutant (Mut) vector and transfected into PDAC cells for 48 h (Figure S1D). We found that overexpressing WT and 1503–91aa ORF vectors tagged with Flag could be translated into microproteins through Western blotting, whereas overexpression with Mut vectors could not, suggesting that 1503–91aa was present in the PDAC cells (Figures 1F and S1E). We found that the expression of 1503–91aa was elevated in drug‐resistant pancreatic cancer cell lines compared to that in WT cells (Figures 1G and S1F). Subsequently, we confirmed that GEM treatment increased 1503–91aa expression in PDAC‐resistant cells in a dose‐dependent manner (Figures 1H and S1G). We constructed two PDX PDAC models; GEM‐resistant and GEM‐sensitive. The results showed that the expression level of 1503–91aa was markedly elevated in GEM‐resistant PDX compared with that in sensitive PDX (Figure 1I,J). Needle biopsy specimens from PDAC patients were collected prior to GEM‐based chemotherapy, and their response to chemotherapy was followed up. Using immunofluorescence (IF) staining, we observed that 1503–91aa was significantly elevated in chemotherapy‐resistant tissue specimens compared to chemotherapy‐sensitive specimens, whereas the levels of 1503–91aa were upregulated in cancer tissues compared with adjacent non‐tumour tissues (Figures 1K‒M and S1H). Moreover, high 1503–91aa expression in PDAC tumours correlated with an unfavourable prognosis (Figure 1N).

### 3.2 CTCF increases the expression of LINC01503 and 1503–91aa in PDAC

**FIGURE 2:**
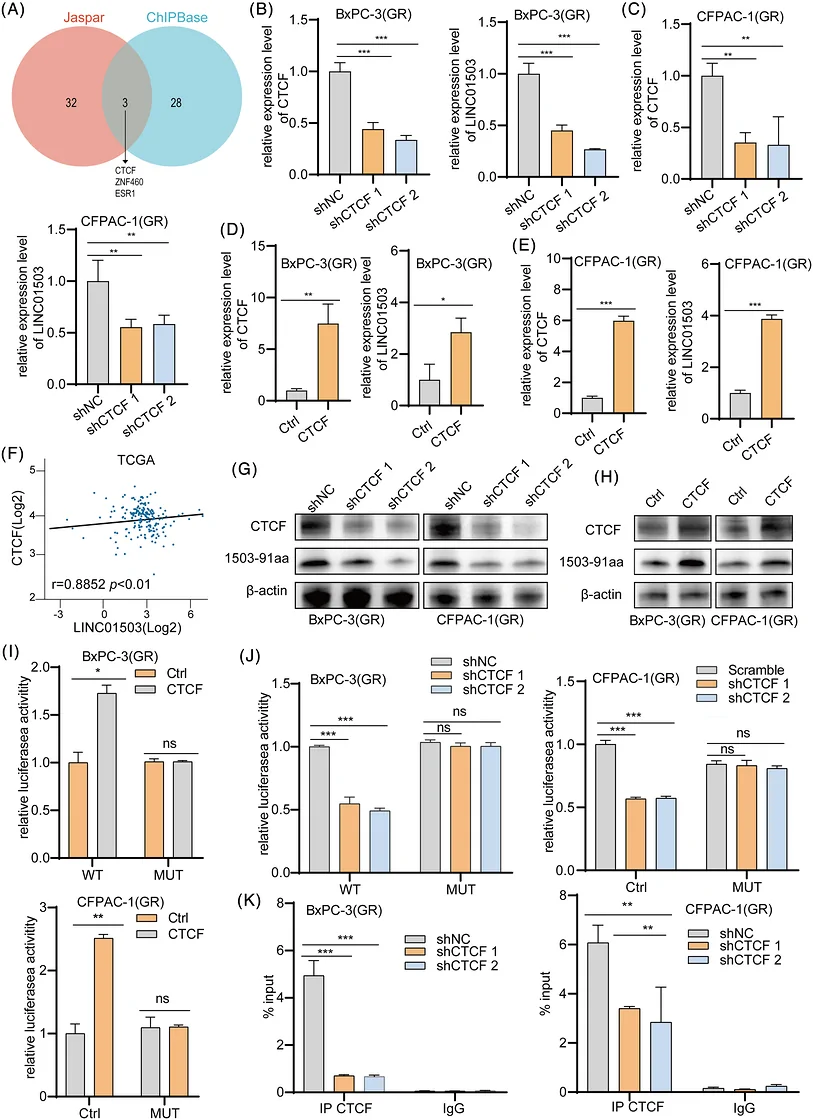
CTCF increases LINC01503 expression and the 1503–91aa in pancreatic ductal adenocarcinoma (PDAC) cells. (A) Transcription factors potentially binding to LINC01503 as predicted by databases. (B and C) Detection of LINC01503 expression in PDAC cells after knockdown of CTCF. (D and E) Detection the expression level of LINC01503 after overexpression of CTCF. (F) Correlation between LINC01503 and CTCF mRNA expression (TCGA dataset). (G and H) Detection of 1503–91aa protein expression in PDAC cells after overexpression or knockdown of CTCF. (I and J) Relative luciferase activities were determined the promoter of LINC01503 with CTCF. (K) Chromatin immunoprecipitation (ChIP)‐qPCR assay to verify assess CTCF‐binding levels at the promoter of LINC01503 in in CTCF‐KD and control gemcitabine‐resistant PDAC cells. The data are shown as mean ± SD. ^*^ *p* < .05; ^**^ *p* < .01; ^***^ *p* < .001.

To unravel the potential mechanism underlying 1503–91aa dysregulation in GEM‐resistant PDAC, we examined the possible sequences of the LINC01503 promoter and identified potential transcription factors with the LINC01503 promoter in the databases (Figure 2A). After overexpressing ESR1 and ZNF460, we performed RT‐qPCR and observed no significant change in the expression level of LINC01503 (Figure S2A,B). Subsequently, we found that ESR1 and ZNF460 levels did not increase in pancreatic cancer (Figure S2C,D). To further investigate the role of CTCF, we designed knockdown (KD) vectors and corresponding negative controls. We verified the KD efficiency and observed that the expression of LINC01503 was significantly reduced in PDAC cells (Figure 2B,C). Conversely, we constructed CTCF overexpression vectors, resulting in a significant upregulation of LINC01503 (Figure 2D,E). Additionally, analysis of TCGA datasets revealed that CTCF expression is upregulated in PDAC and is positively correlated with LINC01503 expression (Figures 2F and S2G). We further detected the corresponding protein levels and found that the 1503–91aa expression patterns were consistent with the LINC01503 transcript levels (Figures 2G,H and S2E,F).

To explore whether CTCF directly binds to the promoter region of LINC01503 to modulate the transcription and expression of LINC01503, we utilised the hTFtarget database to predict potential binding sites between CTCF and LINC01503. Subsequently, we constructed luciferase reporter plasmids containing both WT and mutated CTCF‐binding sites of LINC01503. By comparing the relative luciferase activity in BxPC‐3(GR) and CFPAC‐1(GR) cells, we found that the overexpression and KD of CTCF had a discernible impact on luciferase activity in the WT group but not in the mut‐type group (Figure 2I,J). Moreover, chromatin immunoprecipitation (ChIP) assay results indicated that the promoter region of LINC01503 was enriched in CTCF‐binding signals, and the KD of CTCF significantly decreased the enrichment of CTCF (Figure 2K).

### 3.3 Depletion of 1503–91aa enhances the sensitivity of PDAC‐resistant cells to GEM

**FIGURE 3:**
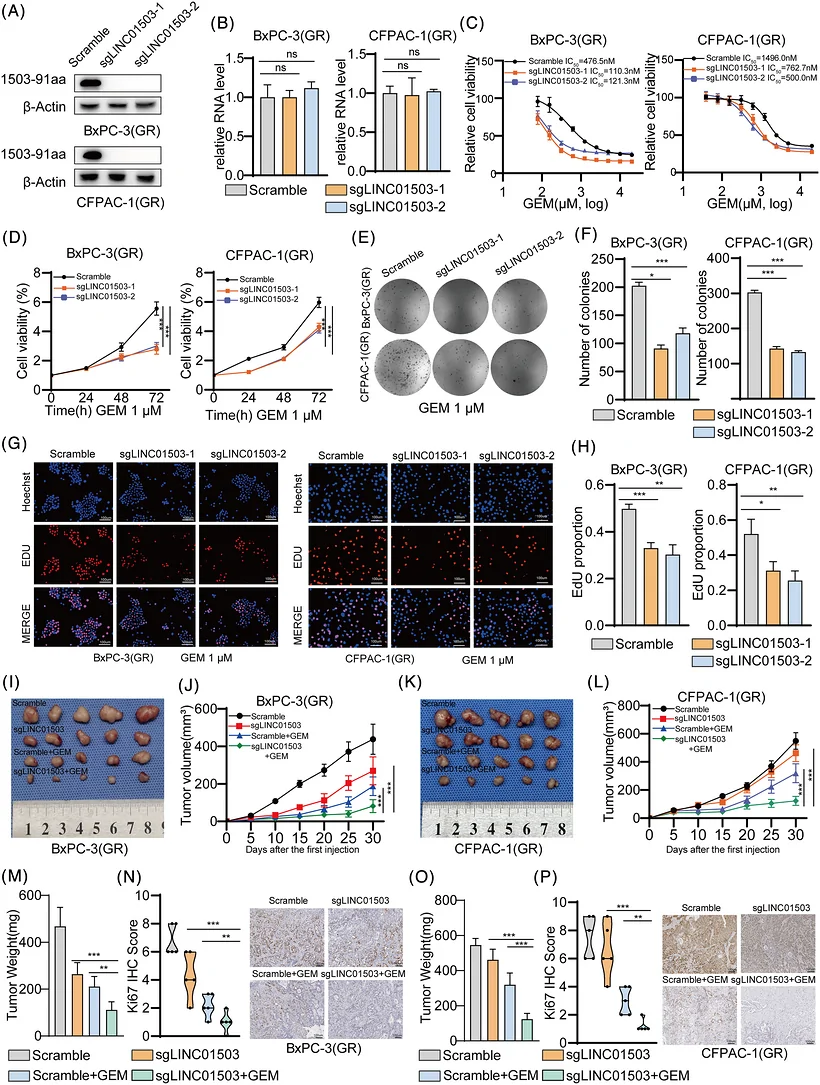
1503–91aa promotes gemcitabine (GEM) resistance in pancreatic ductal adenocarcinoma (PDAC) both in vitro and in vivo. (A and B) 1503–91aa protein and LINC01503 RNA levels in PDAC cell lines transfected with sgLINC01503. (C) Half maximal inhibitory concentration (IC_50_) value changes after knocking out 1503–91aa. (D) Cell‐Counting Kit‐8 (CCK‐8) assays were used to detect the viability of the PDAC cells after transfection with sgLINC01503 or control and treated with 1 µM GEM. (E and F) Colony formation assays were used to detect the viability of the PDAC cells after transfection with sgLINC01503 or control and treated with 1 µM GEM. Statistical analysis of colony formation assays in BxPC‐3(gemcitabine‐resistant, GR) and CFPAC‐1(GR) cells. (G and H) Proliferation abilities are detected using EdU assays and treated with 1 µM GEM. (I and K) Xenograft tumours derived from BxPC‐3(GR) and CFPAC‐1(GR) cells were shown. (J and L) Tumour growth curves after the injection of sgLINC01503 and control of GEM‐resistant cells. (M and N) Tumour weight, statistical analysis and representative images of immunohistochemistry (IHC) staining of Ki67of each BxPC‐3(GR) group. (O and P) Tumour weight, statistical analysis and representative images of IHC staining of Ki67of each CFPAC‐1(GR) group. The data are shown as mean ± SD. ^*^ *p* < .05; ^**^ *p* < .01; ^***^ *p* < .001; ns, not significant, according to Student's *t*‐test.

To investigate whether LINC01503‐encoded protein influences the effect of GEM resistance in PDAC, we knocked out 1503–91aa in GEM‐resistant PDAC cells. 1503–91aa knockout mediated by sgRNA did not affect LINC01503 expression but significantly repressed 1503–91aa expression in PDAC cells (Figures 3A,B and S3A,B). Next, we determined that the half‐maximal inhibitory concentration IC_50_ values indicated that the 1503–91aa knockout significantly reduced the IC_50_ concentration of PDAC GEM‐resistant cells (Figure 3C). Furthermore, under treatment with specific concentrations of GEM, the 1503–91aa deficiency significantly inhibited the proliferation of GEM‐resistant cells (Figure 3D). Additionally, plate colony formation assays demonstrated that 1503–91aa knockout impaired the colony formation ability of GEM‐resistant PDAC cells (Figure 3E,F). Moreover, EdU assays confirmed these results (Figure 3G,H). To evaluate the effect of 1503–91aa on GEM resistance in vivo, BxPC‐3(GR) and CFPAC‐1(GR) cells that stably knockout 1503–91aa were subcutaneously injected into mice. After 30 days of treatment, the 1503–91aa‐knockout group showed a significant reduction in tumour volume and weight after GEM treatment (Figure 3I–M,O). In addition, there was no significant difference in body weight among the four groups (Figure S3C). Immunohistochemistry (IHC) staining revealed that Ki67 was the least expressed in the GEM‐treated group after 1503–91aa knockout (Figure 3N,P). Taken together, these results suggest 1503–91aa deficiency enhances the antitumour effect of GEM. To further exclude the possibility that LINC01503, but not 1503–91aa, plays a role in GEM resistance in PDAC, we established stable LINC01503‐KD PDAC cells via shRNA transfection. We restored the protein expression of 1503–91aa (ORF) in LINC01503 stable PDAC KD cells (Figure S4A). Rescue experiments showed that re‐expression of 1503–91aa in LINC01503‐KD cells rescued PDAC resistance to GEM and most malignant phenotypes (Figure S4B–G), suggesting that 1503–91aa but not LINC01503 is the major executor of resistance in PDAC.

### 3.4 1503–91aa interacts with CPT1A by the site of Arg243

**FIGURE 4:**
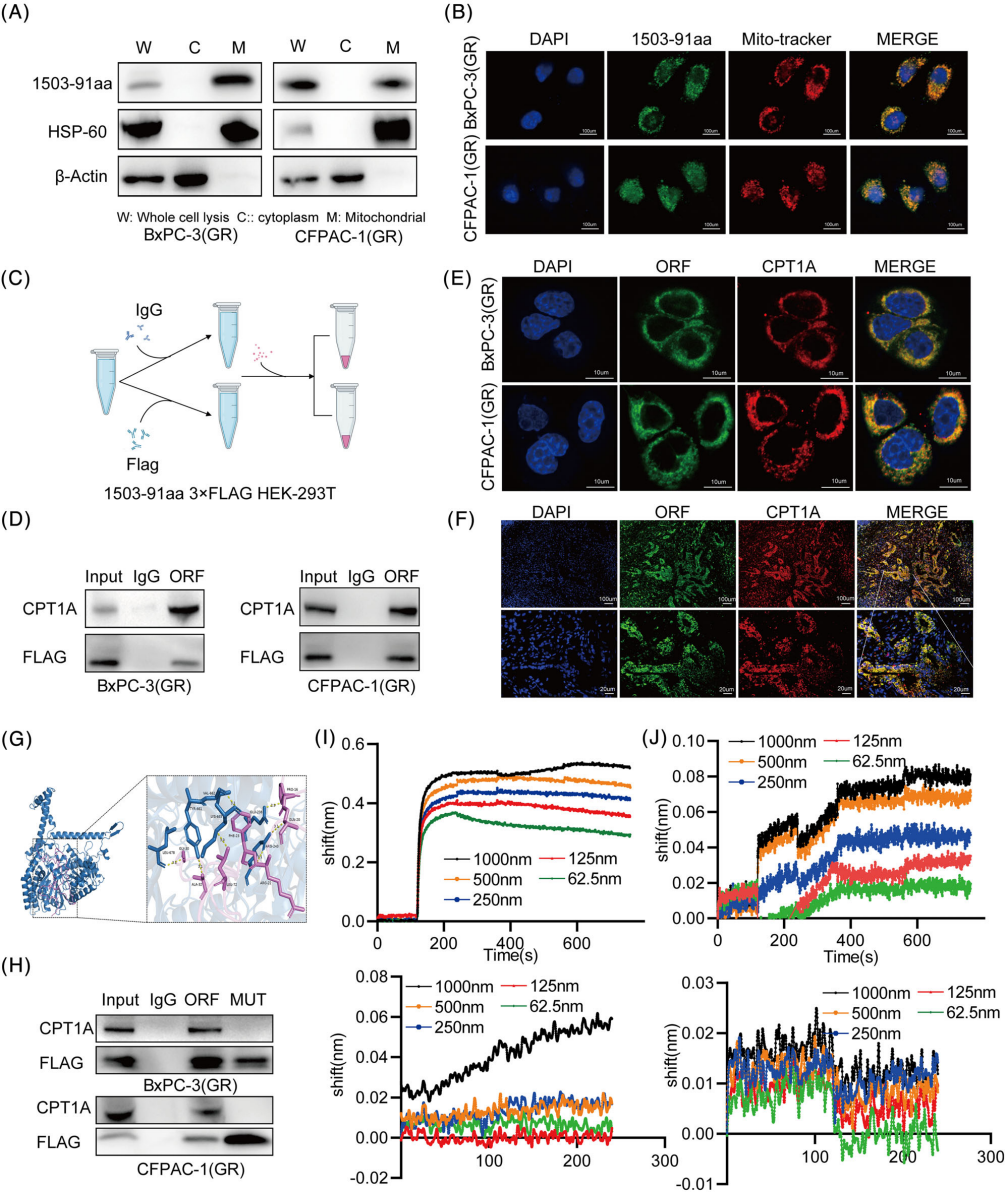
1503–91aa binds to carnitine palmitoyl transferase 1A (CPT1A) and increases its activity by competitively inhibiting malonyl‐CoA (MCoA). (A) Mitochondrial fractionation assays confirmed the mitochondrial localisation of 1503–91aa. (B) Immunofluorescence (IF) confirmed the mitochondrial localisation of 1503–91aa. (C) Schematic diagram of co‐immunoprecipitation (co‐IP) combined with LC‒MS/MS to screen potential proteins interacted with 1503–91aa. (D) The interaction between 1503–91aa and CPT1A is confirmed in two pancreatic ductal adenocarcinoma (PDAC) cell lines by co‐IP. (E and F) The co‐localisation of 1503–91aa and CPT1A in BxPC‐3(gemcitabine‐resistant, GR) and CFPAC‐1(GR) cells. (G) Molecular docking simulation of 1503–91aa and CPT1A performed by AutoDock and predicted key amino acids mediating 1503–91aa/CPT1A interaction. (H) co‐IP for verifying the interaction between mutant1503‒91aa and CPT1A. (I and J) Biolayer interferometry (BLI) analysis of the interaction between 1503–91aa and CPT1A/CPT1A mut Arg243. The data are shown as mean ± SD. ^*^ *p* < .05; ^**^ *p* < .01; ^***^ *p* < .001.

To further validate the subcellular localisation of 1503–91aa, we performed mitochondrial fractionation assays, which revealed that 1503–91aa was predominantly localised in the mitochondria (Figures 4A and S5A). IF co‐localisation analysis also confirmed the localisation of 1503–91aa (Figure 4B). For the mechanistic study of 1503‒91aa, we performed IP combined with MS and analysed its potential binding partners (Figures 4C and S5B). In total, 121 proteins were identified from the immunoprecipitants pulled down with 1503–91aa. Combining the mitochondrial localisation of 1503–91aa in cells with gene and functional analysis from the MS results (Figure S5C), we found that CPT1A, a protein reported to be predominantly localised in mitochondria, might potentially interact with 1503–91aa. The interaction between 1503–91aa and CPT1A was further confirmed by IP in PDAC cells (Figures 4D and S5D). In addition, using IF assays, we demonstrated that 1503–91aa co‐localised with CPT1A (Figure 4E,F). Molecular docking analysis, which prioritises conformations with the lowest binding energy and highest cluster frequency, identified aa 20 of 1503–91aa and aa 243 of CPT1A as the key interacting residues (Figure 4G). Arg243, a residue completely conserved across all CPT1 members, is critical for malonyl‐CoA (MCoA) binding and is the best‐known physiological inhibitor of CPT1A.^31^, ^32^ We generated a MUT of the 91aa protein by introducing point mutations into the predicted CPT1A‐binding sites. Following this, we carried out co‐IP assay and found that the mutation of 1503–91aa significantly inhibited its binding to CPT1A (Figures 4H and S5E). Next, we tested the binding between the purified 1503–91aa protein and CPT1A using biolayer interferometry. 1503–91aa binds to CPT1A with remarkably high affinity (Figure 4I). Mutating Arg243 in CPT1A resulted in a drastic reduction in the 1503–91aa association with CPT1A (Figure 4J). The above experiment showed that Arg243 is required for 1503–91aa to bind to CPT1A.

### 3.5 CPT1A stabilises CTCF via succinylation to maintain a CTCF/LINC01503‒1503‒91aa positive feedback loop promoting PDAC chemoresistance

**FIGURE 5:**
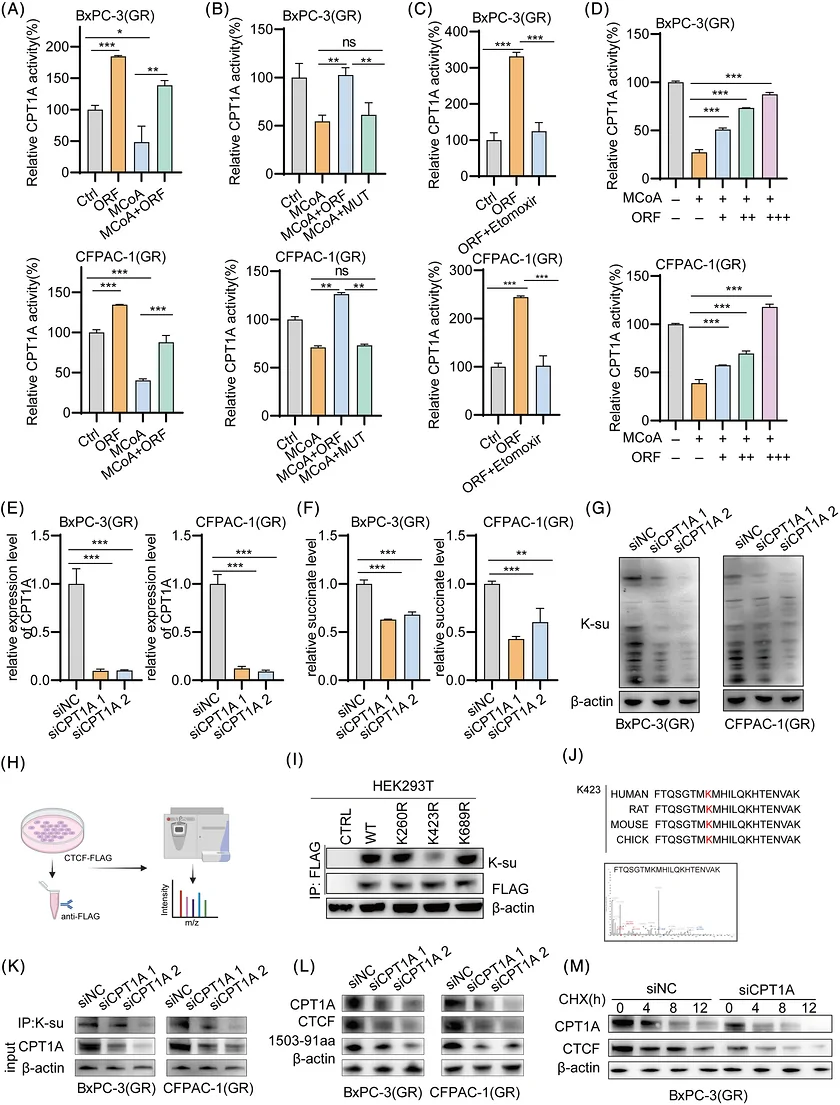
Carnitine palmitoyl transferase 1A (CPT1A) stabilises CTCF through succinylation to maintain the CTCF/LINC01503‑91aa‐positive feedback loop. (A) Relative CPT1A activity was measured in different treatment groups as indicated. Ctrl, control group; ORF, 1503–91aa; MCoA, malonyl‐CoA treatment; and MCoA + ORF, co‐treatment with MCoA and the ORF. (B) ORF, but not MUT, rescues CPT1A activity from MCoA‐mediated inhibition. (C) Etomoxir (Eto) blocked 1503–91aa mediated upregulation in CPT1A activity. (D) Effect of 1503–91aa on MCoA‐mediated CPT1A inhibition. (E) The mRNA level of pancreatic ductal adenocarcinoma (PDAC) cells after CPT1A silencing confirmed by RT‐qPCR. (F) Succinate levels in CPT1A knockdown stable cell lines and wild‐type (WT) cell lines. (G) The succinylation of proteins was detected in WT or CPT1A knockdown stable cell lines. (H) Enrichment of CTCF protein for mass spectrometry identification of its succinylation sites. (I) CTCF succinylation mutant plasmids were expressed in HEK293T cells to examine CTCF lysine succinylation levels. (J) Mass spectrum of K423 succinylation on CTCF and cross‐species conservation of CTCF K423. (K) Western blotting was used to detect the succinylation of the CTCF proteins in WT or CPT1A silencing cell lines. (L) The expression levels of CTCF proteins in WT or CPT1A silencing cell lines were detected by Western blotting. (M) The cells were treated with cycloheximide (CHX) and collected at the indicated time. The degradation rate of the CTCF and CPT1A proteins was detected by Western blotting. The data are shown as mean ± SD. ^*^ *p* < .05; ^**^ *p* < .01; ^***^ *p* < .001.

Subsequently, we found that overexpression of 1503–91aa had no effect on the protein level of CPT1A (Figure S5F). Thus, we hypothesise that competing with MCoA for the same site Arg243, 1503–91aa abrogated MCoA‐mediated CPT1A inhibition. Proximity ligation assay was performed to detect the competitive binding of 1503–91aa and MCoA to CPT1A (Figure S5G). In our in vitro assay, the1503‒91aa protein significantly increased CPT1A activity, whereas MCoA, as expected, downregulated CPT1A activity (Figure 5A). Moreover, the overexpression of 1503–91aa rescued CPT1A activity from MCoA‐mediated inhibition. In contrast, a mutant of 1503–91aa, which is unable to bind to the Arg243 site of CPT1A, had no effect on CPT1A activity (Figure 5B). At the same time, etomoxir blocked the 1503–91aa‐mediated upregulation of CPT1A activity (Figure 5C). To validate our hypothesis that 1503–91aa modulates CPT1A activity by competing for the MCoA‐binding site, we performed an in vitro assay with the purified catalytic domain of CPT1A and found that 1503–91aa counteracted MCoA‐mediated inhibition of CPT1A in a dose‐dependent manner (Figure 5D). Taken together, these results demonstrate that 1503–91aa regulates CPT1A activity by competing with MCoA. Recent studies have revealed that CPT1A has LSTase activity and can promote succinylation modification in cells.^15^, ^16^, ^33^ Therefore, we speculated that CPT1A may regulate mitochondrial function and metabolic reprogramming through succinylation. We subsequently silenced CPT1A expression in CFPAC‐1 and BXPC‐3 cells using siRNA to determine changes in the biological behaviour of PDAC cells (Figure 5E). We measured the levels of succinic acid, a metabolite downstream of CPT1A, and observed markedly reduced succinic acid production in CPT1A‐KD PDAC cells (Figure 5F). Next, we examined the effect of CPT1A on succinylation levels of intracellular proteins. The results showed that CPT1A‐KD decreased the succinylation level of intracellular proteins (Figures 5G and S6A). We also identified a large number of succinylation sites on the transcription factor CTCF. We performed succinylation site sequencing following exogenous overexpression of CTCF (Figure 5H). Three succinylation sites were identified on CTCF. We transfected HEK293T cells with WT CTCF and its mutants (K260R, K423R, K689R). Notably, the K423R mutation drastically reduced CTCF succinylation, indicating that K423 serves as the major succinylation site of CTCF (Figures 5I and S6B). Moreover, the K423 residue is evolutionarily conserved across multiple species (Figure 5J). CPT1A elevates the lysine succinylation level of CTCF (Figure S6C). Therefore, we hypothesised that CPT1A regulated CTCF expression via succinylation. We examined the effects of CPT1A on the succinylation of CTCF, and found that CPT1A‐KD reduced the succinylation of CTCF (Figures 5K and S6D). Subsequently, we examined the expression of CTCF in BxPC‐3(GR) and CFPAC‐1(GR) cells with CPT1A‐KD by Western blotting, which showed that CPT1A‐KD decreased CTCF protein expression (Figures 5L and S6E‒G). Protein degradation rates were examined in cells treated with cycloheximide (CHX). The degradation rate of CTCF in the CPT1A‐KD group was much higher than that in the control group (Figures 5M and S6H). At the same time, the protein level of 1503–91aa also decreases with the KD of CPT1A, indicating that there is a positive feedback loop that increases the protein level of 1503–91aa.

### 3.6 1503–91aa confers GEM resistance in pancreatic cancer through CPT1A‐mediated FAO

**FIGURE 6:**
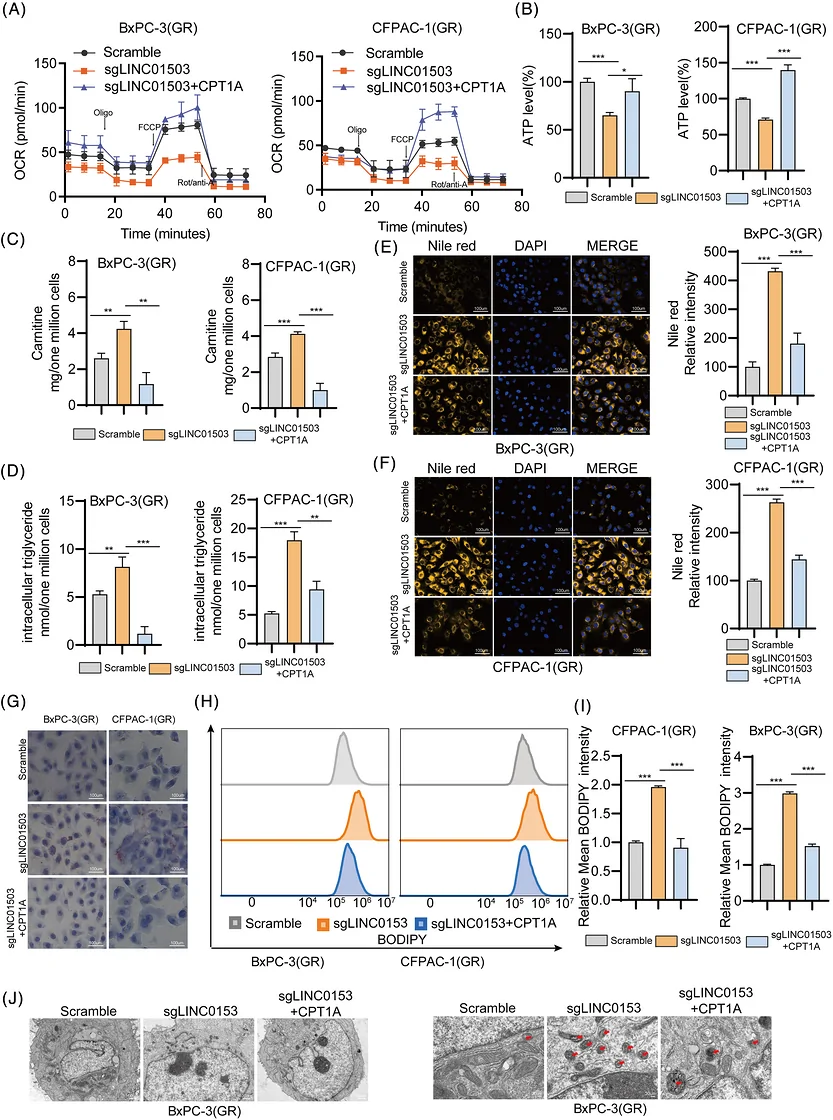
1503–91aa mediates fatty acid oxidation by increasing the activity of carnitine palmitoyl transferase 1A (CPT1A). (A) Pancreatic ductal adenocarcinoma (PDAC) cells were transfected treated with three groups: a control group (Scramble), an LINC01503‐knockdout group (sgLINC01503) and a rescue group where LINC01503 was knocked out with the addition of a CPT1A inhibitor (sgLINC01503 + CPT1A). Oxygen consumption rate (OCR) was measured in real time under basal conditions and in response to oligomycin (1 µM), FCCP (.5 mM) or rotenone/antimycin A (.5 µM) stimulation at the indicated time points. (B) Normalised cellular ATP levels in PDAC cells. (C) Intracellular carnitine content was measured in BxPC‐3(gemcitabine‐resistant, GR) and CFPAC‐1(GR). (D) The intracellular triglyceride level in BxPC‐3(GR) and CFPAC‐1(GR). (E and F) Representative images of Nile red staining and statistical analysis in BxPC‐3(GR) and CFPAC‐1(GR). (G) Cellular neutral lipids were measured in BxPC‐3(WT/GR) and CFPAC‐1(WT/GR) cells by Oil red O staining. (H and I) Flow cytometric analysis of lipid droplets in pancreatic cancer cell lines using BODIPY. (J) Transmission electron microscopy showing mitochondrial morphology. The data are shown as mean ± SD. ^*^ *p* < .05; ^**^ *p* < .01; ^***^ *p* < .001 according to Student's *t*‐test.

In previous studies, CPT1A has been in FAO to regulate the transport of long‐chain fatty acids into the mitochondria for FAO.^34^ To elucidate the role of CPT1A‐mediated FAO in GEM resistance, we performed Seahorse analysis, which revealed that the depletion of 1503–91aa compromised the cellular OCR and ATP generation, while this metabolic defect was rescued by the re‐expression of CPT1A (Figure 6A,B). We found that knockout of 1503–91aa led to a significant increase in the levels of carnitines in the cells, whereas overexpression of CPT1A reversed this effect (Figure 6C).

Consistent with this, due to the impairment of fatty acid catabolism, the levels of lipid droplets and triglycerides in 1503–91aa‐knockout cells also increased significantly. Importantly, increased expression of CPT1A could effectively reverse this phenotype and reduce intracellular lipid accumulation (Figure 6D). Moreover, Nile red and Oil Red O staining demonstrated an increase in lipid droplets in the knockout group compared to the control group; however, this effect was rescued by the overexpression of CPT1A (Figure 6E–G). Furthermore, BODIPY staining, which specifically binds to neutral lipids such as triglycerides, confirmed these findings (Figure 6H,I). Transmission electron microscopy (TEM) imaging was used to visually assess the effect of 1503–91aa knockout on mitochondrial morphology in GEM‐resistant pancreatic cancer cells. BXPC‐3(GR) 1503–91aa‐knockout group exhibited mitochondrial shrinkage and lipid droplet accumulation, suggesting the presence of inhibition of FAO cycle and decrease in cellular homeostasis. Notably, overexpression of CPT1A rescued these morphological defects (Figure 6J). Collectively, 1503–91aa sustains CPT1A activity to maintain fatty acid catabolism. The blockade of this pathway upon 1503–91aa depletion may create a metabolic state that favours chemosensitivity.

**FIGURE 7:**
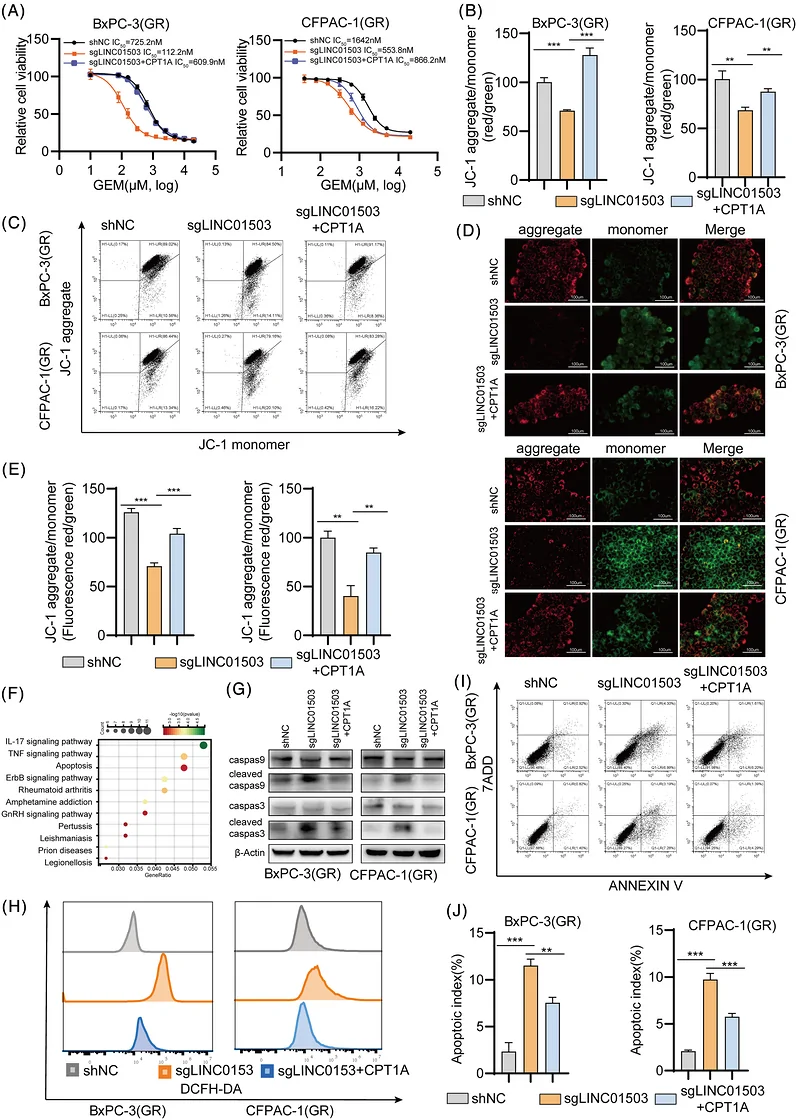
1503–91aa promotes gemcitabine resistance by enhancing carnitine palmitoyl transferase 1A (CPT1A) activity and increasing fatty acid oxidation. (A) Half maximal inhibitory concentration (IC_50_) value of BxPC‐3(gemcitabine‐resistant, GR) and CFPAC‐1(GR) cells treated with three groups: a control group (Scramble), an LINC01503‐knockdout group (sgLINC01503) and a rescue group where LINC01503 was knocked out with the addition of a CPT1A inhibitor (sgLINC01503 + CPT1A). (B and C) Detection of the membrane potential of BxPC‐3(GR) and CFPAC‐1(GR) cells via combined flow cytometry analysis using JC‐1. (D and E) Detection of the membrane potential of BxPC‐3(GR) and CFPAC‐1(GR) cells via immunofluorescence analysis using JC‐1. (F) Kyoto encyclopedia of genes and genomes (KEGG) analysis of RNA sequencing. (G) The caspase3, cleaved caspase3, caspase9 and cleaved caspase9 protein levels after knocked out of 1503–91aa and/or CPT1A. (H) Representative histograms of ROS levels. (I and J) Representative flow cytometry dot plots and statistical analysis of Annexin V/7‐AAD staining for apoptosis detection. The data are shown as mean ± SD. ^*^ *p* < .05; ^**^ *p* < .01; ^***^ *p* < .001.

Subsequently, we performed a GEM IC_50_ assay, which revealed that knocking out 1503–91aa enhanced cellular sensitivity to GEM. Conversely, overexpression restores CPT1A in this context (Figure 7A). Mitochondria are not only the primary site for FAO but also a critical hub for regulating apoptosis.^35^ Therefore, it is highly plausible that the inhibition of FAO resulting from suppressing the 1503–91aa/CPT1A axis may trigger apoptosis by disrupting mitochondrial functional homeostasis. Thus, we conducted a systematic evaluation of mitochondrial function. Under normal conditions, the mitochondria maintain a highly negative membrane potential. The JC‐1 assay showed that the knockout of 1503–91aa caused a significant decrease in MMP, as evidenced by an increase in JC‐1 monomers (green fluorescence) and a decrease in aggregates (red fluorescence). This loss of MMP was effectively rescued by the overexpression of CPT1A in a 1503–91aa‐knockout background (Figure 7B–E).

As a cationic probe, TMRE accumulates in the mitochondrial matrix upon entering the cell and emits bright a red fluorescence. The knockout of 1503–91aa reduced red fluorescence, whereas overexpression of CPT1A restored this effect (Figure S7A‒C). Additionally, RNA sequencing of 1503–91aa‐knockout cells revealed that the apoptosis signaling pathway was significantly upregulated (Figure 7F). We examined the expression of genes involved in the apoptotic. The results showed that the expression level of caspase3 and caspase9 were not significantly changed, whereas the expression level of cleaved caspase3 and cleaved caspase9 were significantly upregulated after 1503–91aa knockout. However, CPT1A overexpression inhibits this activation (Figures 7G and S7D,E). Furthermore, as a typical concomitant event of mitochondrial dysfunction, knockout of 1503–91aa led to a sharp increase in reactive oxygen species (ROS) levels, whereas CPT1A overexpression alleviated this oxidative stress (Figures 7H and S7F‒H). Knockout of 1503–91aa significantly increased the proportion of apoptotic cells, whereas CPT1A overexpression reversed this effect (Figure 7I,J).

As reported previously, H473A eliminates both carnitine palmitoyltransferase (CPTase) and LSTase activities of CPT1A, whereas G710E only ablates lipid metabolic transferase activity while retaining succinylation catalytic function.^15^ WT CPT1A overexpression enhanced CTCF succinylation, whereas H473A/G710E succinylation site mutations reversed this effect in PDAC cells (Figure S8A). OCR detection revealed elevated mitochondrial respiration in WT‐CPT1A‐transfected GEM‐resistant PDAC cells. Consistently, WT CPT1A upregulated intracellular ATP and succinate but reduced triglyceride levels (Figure S8B‒E). JC‐1 staining further validated that intact CPT1A succinylation maintains normal MMP in PDAC cells (Figure S8F,G). In summary, our study demonstrates that inhibiting the 1503–91aa/CPT1A regulatory axis blocks FAO, induces mitochondrial dysfunction (decreased MMP), activates the intrinsic apoptotic pathway, and ultimately restores PDAC GEM sensitivity.

### 3.7 Inhibition etomoxir disrupting the CTCF/LINC01503‒1503‒91aa/CPT1A positive feedback loop can significantly enhance the therapeutic efficacy of GEM against chemoresistant PDAC

**FIGURE 8:**
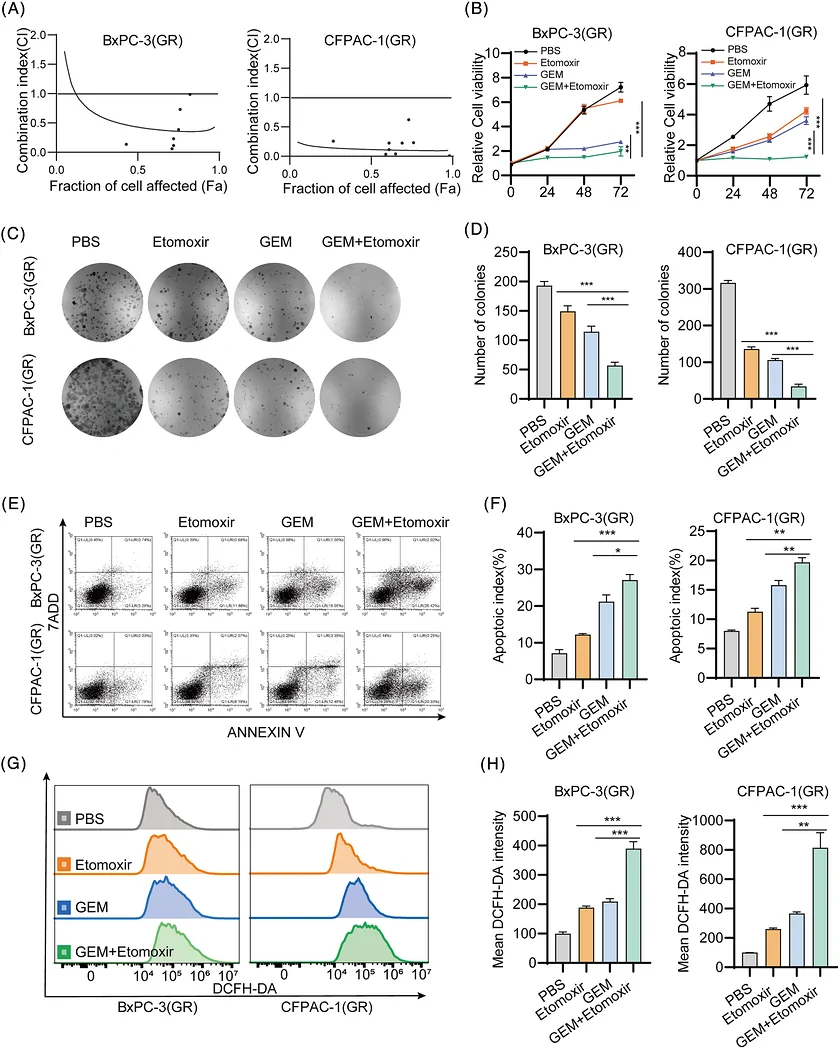
Blocking the CTCF/Linc01503‒1503‒91aa/CPT1A regulatory axis exhibits synergistic therapeutic effects with gemcitabine (GEM) in pancreatic ductal adenocarcinoma (PDAC). (A) The combination index of GEM and etomoxir in BxPC‐3(gemcitabine‐resistant, GR) cells. The combination index of GEM and etomoxir in CFPAC‐1(GR) cells. (B) Cell viability assay of BxPC‐3(GR) and CFPAC‐1(GR) cells in different treatment groups. (C and D) Colony‐formation assay with etomoxir and GEM treatment in GEM‐resistant cells. (E and F) Representative flow cytometry dot plots and statistical analysis of Annexin V/7‐AAD staining for etomoxir and GEM treatment in GEM‐resistant cells. (G and H) Flow cytometry ROS experiment with etomoxir and GEM treatment in different treatment groups. The data are shown as mean ± SD. ^*^ *p* < .05; ^**^ *p* < .01; ^***^ *p* < .001. CPT1A, carnitine palmitoyl transferase 1A.

Taken together, our previous results suggest that CPT1A stabilises CTCF protein levels through succinylation, enabling CTCF to promote the sustained expression of LINC01503/1503‒91aa, which in turn continuously drives FAO and leads to PDAC GEM resistance. Thus, we hypothesised that targeting this positive feedback loop to block FAO might be a novel therapeutic approach in GEM‐resistant PDAC, and we evaluate the feasibility of combining the CTP1A inhibitor etomoxir with the traditional chemotherapy drug GEM. We tested the combination index (CI) of the two drugs in GEM‐resistant cells. The results showed that etomoxir and GEM had a synergistic therapeutic effect when the two drugs were administered simultaneously (Figure 8A). Further evaluating the effect of etomoxir in combination with GEM compared to monotherapy, we conducted cell viability and colony formation. The findings revealed that the combined treatment of etomoxir and GEM more profoundly inhibited the proliferation and colony formation ability of GEM‐resistant cells (Figure 8B–D). Moreover, flow cytometry analysis of apoptotic cells confirmed that the combination therapy significantly increased the number of apoptotic cells in GEM‐resistant cells (Figure 8E,F). Similarly, ROS levels also showed the same trend (Figure 8G,H). These experimental results indicate that the combined therapy of etomoxir with GEM exerts a synergistic therapeutic effect on PDAC.

### 3.8 Blocking the CTCF/LINC01503‒1503‒91aa/CPT1A‐positive feedback loop sensitises PDAC cells to GEM in PDAC PDXs

**FIGURE 9:**
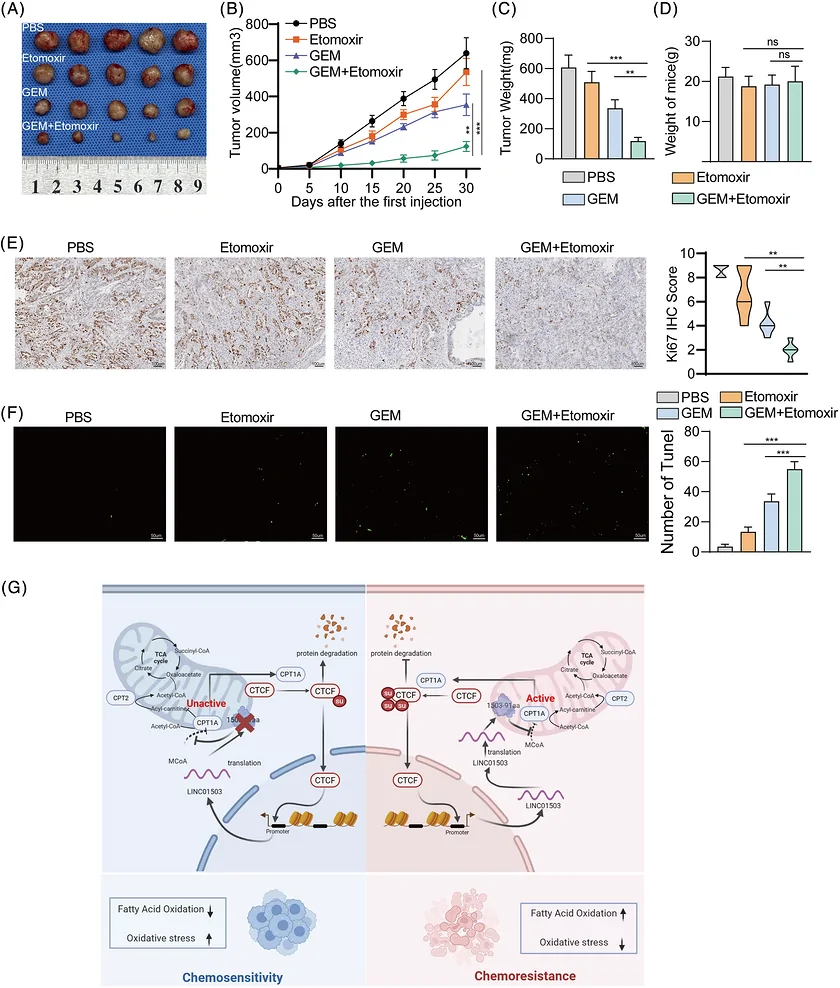
Blocking the CTCF/Linc01503‒1503‒91aa/CPT1A regulatory axis can significantly enhance the therapeutic efficacy of gemcitabine (GEM) against chemoresistant pancreatic ductal adenocarcinoma (PDAC) patient‐derived xenograft (PDX). (A) Representative images of tumours from PBS, etomoxir, PBS, and etomoxir + GEM groups. (B) Tumour growth curves of each group. (C) Tumour weight of each group. (D) Weight of mice. (E) Representative immunohistochemistry (IHC) staining and statistical analysis of Ki67 in tumours with different treatments. (F) Representative images and statistical analysis of TUNEL analysis in tumours with different treatments. (G) Graphical abstract. The data are shown as mean ± SD. ^*^ *p* < .05; ^**^ *p* < .01; ^***^ *p* < .001. CPT1A, carnitine palmitoyl transferase 1A.

We further investigate the therapeutic effect of the etomoxir plus GEM regimen in vivo PDAC PDX model. We treated the PDAC PDXs with etomoxir plus GEM, GEM alone, or etomoxir alone, and closely monitored the tumour growth in different groups of mice. After 30 days of treatment, the combination of etomoxir and GEM strikingly suppressed the proliferation of GEM‐resistant PDX tumours (Figure 9A–C). And the combined treatment had no significant effects on mouse weights, liver and kidney function (Figures 9D and S9A,B). IHC assays revealed the weakest staining of the proliferation marker Ki67 in the combined treatment group (Figure 9E), while the TUNEL assays showed the highest apoptotic rates in this group (Figure 9F). These results imply that the etomoxir may synergistically improve the antitumour effect of GEM without increasing side effects.

## 4 DISCUSSION

Since the concept of precision medicine was first proposed, identifying effective target molecules has become central to cancer treatment.^36^ However, protein‐coding genes account for less than 2% of the total genome, and as research progresses, promising therapeutic targets derived from known protein databases seem to be exhausted.^37^ lncRNAs, which represent most of the human transcriptome and ware once considered transcriptional noise, have emerged as critical regulators of gene expression and are key players in cancer biology.^38^ Based on the development of ribosome profiling (Ribo‐seq), full‐length translating RNA analysis (RNC‐seq) and MS technology, it has been discovered that some lncRNAs have the ability to encode small peptides or microproteins.^39^ Interestingly, researchers discovered that some specific circumstances or stimuli, such as tumourigenesis and heat shock, might trigger a ‘switch’ from the non‐coding to the coding state by modifying ncRNA sequences.^40^ Emerging research suggests that microproteins derived from lncRNAs are pivotal in tumourigenesis and tumour progression.^26^, ^27^, ^41^, ^42^, ^43^, ^44^ These studies suggest that unknown encoded proteins derived from lncRNA may provide more therapeutic strategies for GEM resistance in PDAC.

In this study, we described a novel microproteins, 1503–91aa, which is translated from LINC01503 and mediates GEM resistance in mitochondria. Interestingly, some peptides encoded by lncRNAs and their physiological roles during cellular respiration, mitochondria metabolism and FAO have been adequately demonstrated.^45^, ^46^, ^47^ Metabolic reprogramming, well‐established hallmark of cancer, in the modification of their metabolism to support the extended energy demand due to their rapid growth and proliferation.^9^ Mitochondrial fatty acid β‐oxidation is the major pathway for the catabolism of fatty acids, and it plays an essential role in maintaining whole body energy homeostasis.^48^ Tumour‐intrinsic FAO may impact on metabolic cues and immune environment in vivo.^49^ Research findings revealed metformin remodelling metabolic reprogramming has potential to convert patients resistant to immunotherapy into those that receive clinical benefit.^50^ In addition to energy production for supporting tumour growth, FAO activation has been implicated in cancer resistance to certain drugs.^51^, ^52^, ^53^, ^54^

CPT1 constitutes a rate‐limiting step of FAO by shuttling long‐chain fatty acids into the mitochondria. CPT1A is a subtype of the CPT1 transport system that, controls the entry of fatty acids into mitochondria for oxidation.^14^, ^55^ In mitochondrial FAO pathways, targeting CPT1A generates clinical benefits in radiation therapy for breast cancer patients and nasopharyngeal carcinoma.^35^, ^56^ Previous studies demonstrated that CPT1A impairs FAO and increases the sensitivity of cancer cells to immune cytotoxicity, thereby inducing drug resistance.^57^ Meanwhile, researchers have found that combining CPT1A inhibitors with carboplatin reduces ovarian cancer growth.^58^ These studies suggest that CPT1A could play a role in making cancer cells resistant to drugs by facilitating FAO. Previous studies have reported that BCoA competed with MCoA, the best‐known metabolic intermediate inhibiting CPT1A, to unleash CPT1A activity for FAO.^32^

In our study, we found that 1503‒91aa binds to CPT1A through Arg243, thereby competing for the binding of MCoA to CPT1A, which ultimately enhance CPT1A activity. 1503–91aa promotes GEM resistance by enhancing CPT1A‐mediated FAO. This process, driven by the 1503–91aa/CPT1A axis, efficiently catabolises fatty acids into acetyl‐CoA and ATP. These products then fuel the electron transport chain, which is essential for maintaining MMP stability. Simultaneously, this process reduces the accumulation of lipid droplets and neutral fats, thereby preventing the excessive production of oxidative stress factors within cells. A stable MMP prevents mitochondrial outer membrane permeabilisation, thereby inhibiting the intrinsic apoptotic cascade and allowing cancer cells to survive chemotherapy. Together, the preservation of a stable MMP and the reduction in oxidative stress create a robust cellular environment that prevents mitochondrial outer membrane permeabilisation, thereby inhibiting the intrinsic apoptotic cascade and allowing cancer cells to survive chemotherapy. CPT1A is highly expressed in various cancers and plays a crucial role in tumour development. It has two types of enzymatic activities: CPTase and LSTase.^14^, ^15^ CPTase activity promotes the degradation of long‐chain fatty acids, whereas LSTase activity modifies lysine residues of proteins. CPT1A's role in promoting FAO to maintain stemness in CSCs through CPTase activity has been reported.^59^, ^60^ Additionally, its LSTase activity promotes succinylation modification, affecting gene expression and disease development.^15^, ^16^ However, the relationship between CPT1A LSTase activity, succinylation modification and stemness maintenance in stem cells requires further exploration. CPT1A has been found to enhance the invasive ability of gastric cancer cells by promoting succinylation modification of S100A10.^16^ We identified CPT1A as a functional LSTase that directly catalyses CTCF succinylation. This modification markedly increased its protein stability. Consequently, a positive feedback loop was established; CTCF promoted LINC01503 and 1503–91aa expression, which activated CPT1A, and CPT1A subsequently stabilises CTCF via succinylation. The CTCF/LINC01503‒1503‒91aa/CPT1A axis sustains malignant phenotype and metabolic reprogramming. Targeting this succinylation‐dependent loop with the CPT1A inhibitor, etomoxir, disrupted cellular FAO levels and synergised with GEM to induce apoptosis. Accumulating reports indicate that combinatorial targeted therapy represents an emerging treatment strategy. A microprotein N1DARP encoded by LINC00261 promotes N1ICD degradation. Targeting the N1DARP–N1ICD interaction represents a promising therapeutic strategy for chemoresistance in pancreatic cancer.^26^ LINC00982 encodes a micropeptide PRDM16‐DT, and its homologous Cimicifugoside H‐1 can alleviate cancer chemoresistance.^61^ Our study illustrates a novel microprotein, encoded by a previously annotated non‐coding RNA, that drives GEM resistance in PDAC. Targeting the 1503–91aa‐mediated FAO axis is worth being developed as a chemotherapeutic adjuvant to the treatment of PDAC, especially for those patients with aberrantly high 1503–91aa expression. And these findings are expected to translate into clinical practice.

## AUTHOR CONTRIBUTIONS

Xiao‐Yu Yin conceived, designed, supervised and guaranteed the study and edited manuscript. De‐Liang Fang performed experiments, analysed the results and drafted the original draft. Si‐Yuan Lu performed the experiments and data curation. Zhi‐De Liu performed the experiments and analysed the results. Qiong‐Cong Xu edited the manuscript and supervised the study. Yang‐Yinhui Yu performed data visualisation and provided resources. Ying‐Qin Zhu curated the data and performed the formal analysis. Ming‐Jian Ma reviewed and edited the manuscript. Jing‐Yuan Ye developed the methodology. De‐Liang Fang, Si‐Yuan Lu, Zhi‐De Liu and Qiong‐Cong Xu contributed equally to this work.

## CONFLICT OF INTEREST STATEMENT

The authors declare they have no conflicts of interest.

## ETHICS STATEMENT

In this study, the human materials were obtained with the consent of patients and approved by the Medical Ethics Committee of the First Affiliated Hospital of Sun Yat‐Sen University (IIT‐2021‐719). All animal procedures were approved by the Animal Care and Use Ethics Committee of Sun Yat‐Sen University, with endeavours made to minimise animal suffering.

## Supporting information



Supporting information

Supporting information

## Data Availability

The data that support the findings of this study are available from the corresponding author upon reasonable request.
