# Supplementary material for: CPT1A through succinylation regulates the CTCF/LINC01503‒91aa feedback loop to promote gemcitabine resistance in pancreatic ductal adenocarcinoma
Source: Clin Transl Med. 2026 Sep 1;16(9):e70776. doi: 10.1002/ctm2.70776 (PMC13535135; doi:10.1002/ctm2.70776)
Supplement: Supplementary file 1 [file CTM2-16-e70776-s001.docx]

**Supplementary Tables**

**Table S1. The primary antibodies of our study.**

| **Antibodies** | **Dilution; Manufacturer** |
| --- | --- |
| 1530-91aa | 1:1000 (WB), 1:100 (IHC), 1:200 (IF); GenScript, China |
| FLAG | 1:1000 (WB)，1:200 (IF); Proteintech, China |
| CPT1A | 1:1000 (WB)，1:200 (IF); Proteintech, China |
| caspase9 | 1:1000 (WB), 1:200 (IHC); CST, USA |
| cleaved caspase9 | 1:1000 (WB), 1:200 (IHC); CST, USA |
| caspase3 | 1:1000 (WB); CST, USA |
| cleaved caspase3 | 1:1000 (WB), 1:200 (IHC); CST, USA |
| CTCF | 1:1000(WB); ABclonal, China |
| Pan-Succinyl-K | 1:1000(WB); ABclonal, China |
| β-Actin | 1:3000 (WB); Proteintech, China |
| HSP-60 | 1:1000 (WB); CST, USA |

**Table S2. Sequences used for experiments in this study.**

| **Primers** | |
| --- | --- |
| LINC01503-F | GGGACGGAGACAAATGACGG |
| LINC01503-R | GGCCTGACACGTAGGTACAC |
| CTCF-F | GAATTGGTTCGGCATCGTCG |
| CTCF-R | GGCGTAATCGCACATGGAAC |
| ESR1-F | CCTCCTCATCCTCTCCCACA |
| ESR1-R | ATGCGATGAAGTAGAGCCCG |
| ZNF460-F | CTCATTCGACACTTCAACATCC |
| ZNF460-R | GTGGATGCTAAAGTGTCGAATC |
| CPT1A-F | GAAGGTGAAGGTCGGAGTCAACG |
| CPT1A-R | TGCCATGGGTGGAATCATATTGG |
| GAPDH-F | GAAGGTGAAGGTCGGAGTCAACG |
| GAPDH-R | TGCCATGGGTGGAATCATATTGG |
| **Sequences of sgRNA, shRNA and siRNA** |  |
| Scramble | GTATTACTGATATTGGTGGG |
| sgLINC01503 | AGCCTGCTCACATGACCGTG |
| shNC | CCTAAGGTTAAGTCGCCCTCG |
| shLINC01503 | GCTCGGAATACCCACCTTTCT |
| shCTCF 1 | GCAAGGCAAGAAATGCCGTTA |
| shCTCF 2 | AAAGTTGAAGCCCATTGAT |
| siNC | siN0000001-1-5 |
| si CPT1A 1 | GGATGGGTATGGTCAAGATCT |
| si CPT1A 2 | GGATCTGCTGTATATCCTTCC |
