# Supplementary material for: CPT1A through succinylation regulates the CTCF/LINC01503‒91aa feedback loop to promote gemcitabine resistance in pancreatic ductal adenocarcinoma
Source: Clin Transl Med. 2026 Sep 1;16(9):e70776. doi: 10.1002/ctm2.70776 (PMC13535135; doi:10.1002/ctm2.70776)
Supplement: Supplementary file 2 [file CTM2-16-e70776-s002.docx]

**Supplementary Figures**

**Figure S1 Detection of the expression of the lncRNA-encoded protein 1503-91aa**

A. The ORF finder database and SmPort database contains information about the 1503-91aa protein.

B. Peptide antigen sequence design for antibody production.

C. MS detection of 1503-91aa specific peptide sequences in PDAC cells.

D. Schematic diagram of plasmid construction

E. Quantitative densitometry graph of Western blot results shown in Figure 1F.

F. Quantitative densitometry graph of Western blot results shown in Figure 1G.

G. Quantitative densitometry graph of Western blot results shown in Figure 1H.

H. Statistical analysis of 1503-91aa in gemcitabine-resistant and gemcitabine-sensitive tissues.

**Figure S2 Mechanisms underlying the dysregulated expression of 1503-91aa in pancreatic cancer chemoresistance**

A.The mRNA level of ESR1 and LINC01503 after ESR1 overexpression in PDAC cells.

B.The mRNA level of ZNF460 and LINC01503 after ZEN460 overexpression in PDAC cells.

C-D.The expression level of ESR1and ZNF460 in PDAC and normal tissues.

E. Quantitative densitometry graph of Western blot results shown in Figure 2G.

F. Quantitative densitometry graph of Western blot results shown in Figure 2H.

G.The expression level of CTCF in PDAC and normal tissues.

**Figure S3 1503-91aa, rather than LINC01503, enhances the sensitivity of gemcitabine-resistant PDAC cells to gemcitabine**

A. Sequencing results confirming the indel of 1503-91aa ORF in PDAC cells by CRISPR/Cas9.

B. Quantitative densitometry graph of Western blot results shown in Figure 3A.

C. Weight of mice.

**Figure S4 1503-91aa, rather than LINC01503, enhances the resistance of PDAC cells to gemcitabine.**

A. WB used for LINC01503 knockdown (KD) and/or 1503-91aa (ORF) detection

B. The IC_50_ value changes after LINC01503 knockdown (KD) and/or 1503-91aa (ORF).

C. Effect of LINC01503 knockdown (KD) and/or 1503-91aa (ORF)on the CCK8 assay.

D-E. Effect of LINC01503 knockdown (KD) and/or 1503-91aa (ORF)on the colony formation assay with 1 µM gemcitabine.

F-G. Effect of LINC01503 knockdown (KD) and/or 1503-91aa (ORF)on the EdU assay with 1 µM gemcitabine.

The data are shown as mean ± SD. ∗p < 0.05; ∗∗p < 0.01; ∗∗∗p < 0.001 ns, not significant, cording to Student’s t test. GR, gemcitabine resistant.

**Figure S5 To elucidate the molecular mechanism of 1503-91aa-mediated drug resistance**

A. Quantitative densitometry graph of Western blot results shown in Figure 4A.

B. The image of CO-IP silver staining.

C. KEGG pathways enrichment of DEGs in 1503-91aa overexpression groups compared with1503-91aa deficient groups.

D. Quantitative densitometry graph of Western blot results shown in Figure 4B.

E. Quantitative densitometry graph of Western blot results shown in Figure 4C.

F. Western Blotting was performed to examine the effect of 1503-91aa (ORF) on CPT1A expression.

G. Proximity Ligation Assay (PLA) was performed to detect the competitive binding of 1503-91aa and MCoA to CPT1A

**Figure S6 Statistical quantification results**

A. Quantitative densitometry graph of Western blot results shown in Figure 5G.

B. Quantitative densitometry graph of Western blot results shown in Figure 5I.

C. CTCF in vitro succinyltransferase assay

D. Quantitative densitometry graph of Western blot results shown in Figure 5L.

E-G. Quantitative densitometry graph of Western blot results shown in Figure 5M.

H. Quantitative densitometry graph of Western blot results shown in Figure 5N.

**Figure S7 LINC01503 knockdown induces ROS accumulation, and the effect of CPT1A is cell line-dependent**

A-C. Determination of mitochondrial membrane depolarization by TMRE.

D-E. Quantitative densitometry graph of Western blot results shown in Figure 5H.

F-G. Detection of superoxide in live PDAC cells using MitoSOX and the statistic diagram of superoxide in live PDAC cells using MitoSOX.

H. The statistic diagram of the intracellular ROS levels measured by DCFH-DA fluorescence intensity in BxPC-3(GR) and CFPAC-1(GR) cells

Figure S8. CPT1A succinylation mutation regulates CTCF、lipid metabolism and mitochondrial function in PDAC cells

A. Detecting CTCF–CPT1A binding and succinylation of CTCF with different CPT1A mutants.

B: OCR curves of BxPC-3 and CFPAC-1 cells expressing Vector, H473A, G710E or wild-type (WT) CPT1A.

C. Relative intracellular ATP levels in PDAC cell lines with indicated CPT1A constructs.

D. Relative cellular succinate abundance under different CPT1A status

E. Relative intracellular triglyceride contents in PDAC cells

F-G. JC-1 staining and quantitative statistics for mitochondrial membrane potential (MMP)

**Figure S9 Toxicity assessment of drug combination**

A. H&E staining analysis of mouse liver

B. Evaluation of nephrotoxicity in mice.
